# Retrospective Analysis of the Effect of Postmenopausal Women Medications on SARS-CoV-2 Infection Progression

**DOI:** 10.3390/life14091107

**Published:** 2024-09-03

**Authors:** Veronica Cocetta, Manuel Zorzi, Stefano Bejor, Maria Candida Cesta, Maria De Pizzol, Jean-Philippe Theurillat, Marcello Allegretti, Andrea Alimonti, Monica Montopoli, Massimo Rugge

**Affiliations:** 1 Department of Pharmaceutical and Pharmacological Sciences, University of Padova, 35122 Padova, Italy; veronica.cocetta@unipd.it (V.C.); stefano.bejor@unipd.it (S.B.); 2 Veneto Tumour Registry, Azienda Zero, 35131 Padova, Italy; manuel.zorzi@azero.veneto.it; 3 Dompé Farmaceutici S.p.A, 67100 L’Aquila, Italy; candida.cesta@dompe.com (M.C.C.); marcello.allegretti@dompe.com (M.A.); 4 Dompé Farmaceutici S.p.A, 20122 Milano, Italy; mariadepizzol@gmail.com; 5 Institute of Oncology Research (IOR), Oncology Institute of Southern Switzerland, 6500 Bellinzona & Università della Svizzera Italiana (USI), 6900 Lugano, Switzerland; jean-philippe.theurillat@ior.usi.ch (J.-P.T.); andrea.alimonti@ior.usi.ch (A.A.); 6 VIMM-Veneto Institute of Molecular Medicine, Foundation for Advanced Biomedical Research, 35129 Padova, Italy; 7 Department of Medicine, University of Padova, 35122 Padova, Italy; 8 Department of Health Sciences and Technology, ETH Zürich, 8092 Zurich, Switzerland

**Keywords:** Sars-CoV-2, vitamin D, osteoporosis, menopausal symptoms, SERM

## Abstract

Since the beginning of the COVID-19 pandemic, it has been evident that women and young people were less susceptible to severe infections compared to males. In a previous study, we observed a reduced prevalence of SARS-CoV-2 infections in hormonal-driven breast cancer patients undergoing SERM (selective estrogen receptor modulator) therapy with respect to other treatments inhibiting estrogen synthesis. In addition to being used in anticancer therapy, SERMs are also prescribed for postmenopausal osteoporosis prevention and treatment. Therefore, in this study, a retrospective analysis of the clinical outcomes of SARS-CoV-2 infections in a population of women over 50 years who were treated for the management of menopausal symptoms was performed. SARS-CoV-2 infections, hospitalizations, and death rates were evaluated in women residing in the Italian north-eastern Veneto Region who were undergoing treatment with Estrogen Modulators (EMs); Estrogen or Progestin, and their combination (EPs); Bisphosphonates (BIs); or cholecalciferol (vitamin D3) ± calcium supplementation (CC). The final cohort study included 124,393 women, of whom 6412 were found to be SARS-CoV-2 infected (CoV2+ve). The results indicated that only women treated with vitamin D3 alone or in combination with calcium showed a significant reduction in their SARS-CoV-2 infection risk by 26% (OR 0.74; 95%CI 0.60–0.91). On the other hand, an increased risk of hospitalization (OR 2.69; 95%CI 1.77–4.07) was shown for the same treatments. The results highlighted in this work contribute to shedding some light on the widely debated role of vitamin D in the prevention of SARS-CoV-2 infections and the disease’s treatment.

## 1. Introduction

At the end of 2019, a novel coronavirus named SARS-CoV-2 caused a global pandemic of a respiratory illness termed COVID-19 [1,2,3]. A SARS-CoV-2 infection causes respiratory symptoms, which may develop into a mild or severe and life-threatening respiratory disease [3]. It has been observed that young people and females are less susceptible to severe COVID-19 infection outcomes [4]. Many factors, including physiology, genetics, and lifestyle, can concur with the gender differences observed, although a large number of studies in the literature have pointed to the implications of hormones in the infection and progression of the disease [4,5,6,7,8,9]. Estrogen, in addition to its primary functions in the regulation of bone homeostasis and secondary sexual characteristics, also plays a key role in the control of pro-inflammatory signals/pathways by regulating innate and adaptive immune responses [10]. Their activity is mediated by nuclear estrogen receptors (ERs) that act as “hormone-activated transcription factors”. The SARS-CoV-2 spike protein interferes with the signaling pathways of estrogen receptors, probably due to the presence in the SARS-CoV-2 spike protein of an aminoacidic (LxxLL) motif, homologous to the motif of the nuclear coactivator 1 (NCOA1), which functions as a coactivator for nuclear receptors and enhances gene transcription by recruiting additional transcriptional machinery [11]. The downregulation of lung inflammation and the decrease in cytokine release observed following ER activation have been confirmed by clinical and experimental studies, thus suggesting a possible mechanism of action [12]. Estrogen can also play a role in stressful conditions by mediating the vasodilatation of the pulmonary vasculature and ameliorating the outcomes of shock and trauma [13,14,15]. In a recent work, we observed a reduced frequency of SARS-CoV-2 infections in hormonal-driven breast cancer patients undergoing SERM (Selective Estrogen Receptor Modulator) therapy (specifically Tamoxifen, Toremifen, or Fulvestrant) compared to treatments inhibiting estrogen synthesis (like Aromatase inhibitors and Luteinizing-hormone-releasing hormone agonists) [16]. SERMs are able to selectively modulate the activity of estrogen receptors in different tissues. These drugs can act as either estrogen receptor agonists (activators) or antagonists (blockers), depending on the target tissue. SERMs have been previously reported to contribute to the blockade of viral entrances and replication in several viral infections, such as SARS-CoV, MERS-CoV, and Ebola, through nonclassical pathways associated with ERs [17,18,19,20]. The results of our study on a fragile oncological population support the protective role of SERMs in current viral infections and diseases [16], prompting us to explore their possible role in a wider sample of patients in depth. Selective estrogen modulator therapy is also prescribed for postmenopausal osteoporosis prevention and treatment [21,22]. Menopause, i.e., the definitive end of menstruation, is due to follicle consumption, with a consequent lack of estrogen and the end of reproductive functions [23].

During menopause, besides other bothersome symptoms, osteoporosis is the most prevalent problem, inducing low bone mineral density and resulting in bone fragility and an increased risk of fractures [24]. Treatments for postmenopausal women include hormone replacement therapy, Bisphosphonates, the anti-receptor activator of NF-κB ligand antibodies (Denosumab), SERMs, the calcitonin parathyroid hormone (PTH) and PTH-related protein, and supplementation with vitamin D and calcium [21,25,26,27]. At present, the SERMs available for the control of menopause-related symptoms are Raloxifene (RLX) and Bazedoxifene (BZA), which are especially useful for their activity on bones [28,29].

To further explore the hypothesized role of SERMs in modulating SARS-CoV-2 infection and disease, we performed a retrospective study investigating the clinical outcomes of SARS-CoV-2 infections in a population of women over 50 years being treated for the management of menopausal symptoms.

The primary endpoint of this study is to assess the frequency and severity of SARS-CoV-2 infections in terms of hospitalization and death in non-oncological menopausal patients undergoing hormonal replacement therapy.

## 2. Materials and Methods

### 2.1. Study Population

This retrospective study investigated the clinical outcomes of infections from SARS-CoV-2 in a population of women over 50 years old who had never been diagnosed with cancer, resided in the Veneto Region (in the northeast of Italy), and had been tested for SARS-CoV-2. Real-time PCR and next-generation sequencing were used to assess the viral status of infected (CoV2+ve) and uninfected (CoV2-ve) women. For women who tested negative multiple times, only the first test result was considered. If a woman had alternative negative and positive test results, she was recorded as CoV2+ve according to the time of her first positive result. The cohort of subjects was divided into the following groups based on the drug therapies taken within the last 12 months before the first SARS-CoV-2 test.

The following classes of drugs were screened:

- Bisphosphonates (BI): Alendronic Acid, Ibandronic Acid, Zolendronic Acid, and combinations with cholecalciferol.
- Estrogen and Progestinic (EP) combination: Estradiol, Estriol, Medroxyprogesterone, Tibolone, Dienogest, Drospirenone, Norethisterone, and combinations.
- Estrogen Modulators (EMs): Raloxifene and Bazedoxifene.
- Cholecalciferol (vitamin D3) and combinations of vitamin D3 and calcium (CC).

### 2.2. Sources of Study Data

The regional archive of COVID-19 swab tests was used to identify women who tested for SARS-CoV-2 infections from February 22 to 31 July 2020, and to retrieve their SARS-CoV-2 infection status [30]. A link with the database of the Veneto Tumor Registry was performed to exclude women who were diagnosed with cancer at the time of their SARS-CoV-2 test.

Comorbidities were recorded and linked with administrative data (hospital discharge records, outpatient service records, pharmaceutical prescriptions, access to emergency departments, and prescription charge exemptions).

Hospitalization data were obtained from the Regional Archives on Hospital Admissions. Vital statuses for SARS-CoV-2 positives were obtained through the record linkage with the Regional Health Service’s population lists as of 31 July 2020.

Information on drug therapies was obtained through a link with the regional Drug Prescriptions Database, which was available up to 31 December 2020.

### 2.3. Statistical Analysis

Women tested for their SARS-CoV-2 status were initially analyzed in order to identify factors associated with a positive test. We then focused on the cohort of CoV2+ve women to identify the variables related to hospital admissions and deaths. The χ2 test for proportions and the Mann–Whitney test were used to evaluate the association between viral statuses, patients’ demographics, and clinical profiles, and median ages, respectively. Prevalence odds ratios with 95% confidence intervals were computed to study the factors associated with SARS-CoV-2 positivity, hospitalizations, and deaths for ages, comorbidities, and treatments performed in the 12 months before SARS-CoV-2 testing.

Predictors of the study outcomes (CoV2+ve status, hospitalization, and death) were then identified through a multivariable stepwise logistic regression model, setting the entry and exit alpha (type I error) at 0.30 and 0.35, respectively (with the only exception being drug therapy that was forced into the model to estimate the factors associated with death).

The SAS EG v.6.1 (SAS Institute Inc., Cary, NC, USA) statistical package was used for all analyses. All statistical tests were two-tailed. A *p*-value < 0.05 was considered statistically significant.

### 2.4. Ethics

The study was formally approved by the Bioethics Committee of the Veneto Regional Authority (protocol No. 245343/2020).

### 2.5. Data Availability

The dataset used for the analysis will be made available by the authors upon request.

## 3. Results

**Figure 1:**
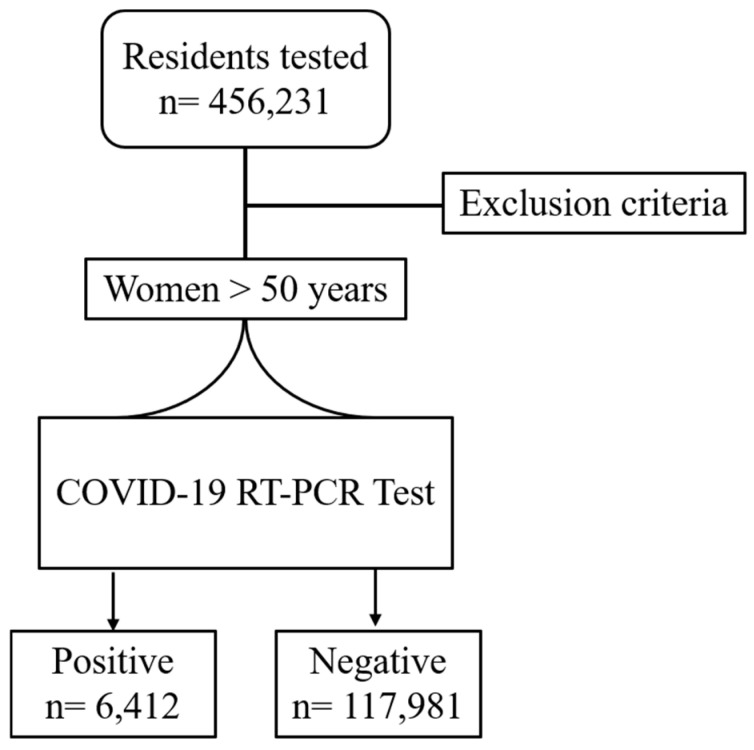
Process flow of patient selection from the global cohort of tested residents (*n* = 456,213) compared to the cohort used for this study.

During the study period, 283,491 out of 456,213 Veneto residents who were tested for SARS-CoV-2 were women (62.1%). Women aged 50 years or more numbered 147,134. After excluding women diagnosed with cancer (*n* = 22,741, 15.5%), the final cohort study included 124,393 women. Among the study population, 6412/124,393 women were found to be CoV2+ve (5.1%) and 117,981 were CoV2-ve (94.9%) (Figure 1).

Table 1 shows the demographics and clinical profiles of the overall study cohort and of CoV2+ve and CoV2-ve women. The median age was 64 years, and this was statistically higher for the CoV2+ve women (75 years) compared to the CoV2-ve women (63 years; *p* < 0.0001). Regardless of SARS-CoV-2 status, the most common comorbidities were cardiovascular (26%), neurologic (8.9%), endocrine (8.0%), respiratory (4.4%) and psychiatric (4.2%). Univariate statistical analysis showed a significantly higher rate of comorbidities in the CoV2+ve female population, particularly for cardiovascular (33.1%), neurologic (12.7%), respiratory (6.2%), psychiatric (5.3%), and renal (3.4%) conditions (all with *p* < 0.0001). There were also significant differences concerning endocrine conditions (CoV2+ve versus CoV2-ve: 8.9% versus 7.9%, respectively; *p* = 0.005). Overall, within the 12 months prior to SARS-CoV-2 testing, 1.9% of women had received CC, and 1.2% had undergone EP treatment. The proportion of women undergoing both CC and EP treatment was higher among CoV2-ve women (respectively 1.9% vs. 1.6% and 1.3% vs. 0.8% in CoV2-ves). Overall, 1537 CoV2+ve women were admitted to the hospital during the follow-up (25%). After a median follow-up of 117 days (interquartile range 101–130 days), 718 women were among those with CoV2+ve who died (11.7%).

Table 2 shows the association between SARS-CoV-2 infections and COVID-19 clinical outcomes, with the explanatory factors selected using the stepwise multivariable model. Age was associated with a slight increase in the risk of infection (OR 1.021; 95%CI 1.019–1.023) and hospitalization (OR 1.018; 95%CI 1.013–1.023) and with a relevant increase in the risk of death (OR 1.095; 95%CI 1.085–1.106). Only neurologic comorbidities significantly modified the risk of infection (OR 1.11; 95%CI 1.02–1.21), while the risks of hospitalization and death were heavily affected by the presence of renal (OR 1.90; 95%CI 1.41–2.57 and OR 1.75; 95%CI 1.24–2.46, respectively), rheumatologic (OR 1.52; 95%CI 1.15–2.01 and OR 1.44; 95%CI 0.96–2.12), and respiratory comorbidities (OR 1.51; 95%CI 1.20–1.90 and OR 1.32; 95%CI 1.01–1.73). (The absolute numbers are reported in Supplementary Table S1).

Compared with women who were not undergoing any of the therapies considered for this study, women treated with estrogen did not show any statistically significant change in their risk of SARS-CoV-2 infection, hospitalization, or death. However, a trend toward lower infection rates and risk of hospitalization is highlighted by the results. Among the other considered treatments, only women treated with CC (cholecalciferol (vitamin D3) and combinations with calcium) showed a significant reduction in the risk of SARS-CoV-2 infection by 26% (OR 0.74; 95%CI 0.60–0.91) and, conversely, an increased risk of hospitalization (OR 2.69; 95%CI 1.77–4.07). The number of events among women undergoing therapy with EMs was too small to allow for an estimate.

## 4. Discussion

Since the beginning of the COVID-19 pandemic, gender disparities in severe cases and higher death rates in male patients have been reported by numerous observations and studies [31,32,33,34].

Collectively, these data indicate the role of sex hormones in both SARS-CoV-2 infection susceptibility and in the COVID-19 disease course. Analogous findings were previously reported for other coronavirus infections, like Severe Acute Respiratory Syndrome (SARS) and the Middle East Respiratory Syndrome (MERS), in which infected patients showed a similar age-dependent susceptibility, sex-related progression, and fatality to disease [35,36,37,38,39]. In our previous study, to validate the hypothesis that hormonal regulation can be implicated in COVID-19 clinical outcomes, we evaluated the prevalence of SARS-CoV-2 infections, hospital admissions, and deaths in women who were affected by hormone-dependent tumors and undergoing treatment with anti-estrogen therapies. In a cohort of 51,060 women tested for SARS-CoV-2 infection, we observed a reduced prevalence of SARS-CoV-2 infections in hormonal-driven patients undergoing SERM therapy but not for treatments that inhibited estrogen synthesis, such as Aromatase Inhibitors (AI) and Luteinizing-hormone-releasing hormone agonists (LAs). Therefore, our study suggests an off-target effect due to SERMs, which potentially involves alterations in the mechanism of fusion between the virus and the host cell [16].

In this work, to further explore the potential role of SERMs in modulating SARS-CoV-2 infection and ongoing disease, we performed a retrospective study investigating the clinical outcomes of SARS-CoV-2 infections in a population of aged women undergoing pharmacological treatments for the management of menopausal symptoms.

In our study, we compared SARS-CoV-2 infections, hospitalizations, and death rates in postmenopausal women undergoing treatment with Estrogen Modulators (EMs); Estrogen, Progestin and their combination (EPs); Bisphosphonates (BIs); and vitamin D3 ± calcium (CC) for the management of symptoms.

Compared to women who were not undergoing any of the therapies considered for this study, women treated with estrogen did not show any statistically significant changes in their risk of SARS-CoV-2 infection, hospitalization, or death. Our study cohort included 124,393 women, and among these, a rather low number of patients received EMs, EPs, BIs, or CC; therefore, looking at the results, no striking effect on infections or hospitalizations was revealed. Nevertheless, our data show a potential protective effect of estrogen, which is open to validation through larger studies.

SERMs are a class of drugs commonly used, especially Raloxifene, which is used to prevent and treat postmenopausal bone fragility [40,41,42,43]. Raloxifene has been reported for its efficacy in handling infectious diseases caused by other RNA viruses, such as Ebola, Influenza A, and Hepatitis C viruses [44]. The results indicate an off-target effect of Raloxifene and, more generally, of SERMs since their antiviral effect is independent of the presence of estrogen receptors [18]. Numerous hypotheses have been formulated on the possible antiviral mechanism of action of Raloxifene, including its interference with viral entry mechanisms at the early stage of infection; its effect on the interaction between the viral envelope and host cell membrane by altering processes of vesicular trafficking; its modulation of the membrane lipidic composition; and the increase in NO levels that occur in the blockage of the viral replication cycle [45]. Furthermore, recently, Raloxifene and Bazedoxifene were found to be capable of interfering with IL-6 signaling in severe COVID-19 patients, showing positive effects on Acute Respiratory Distress Syndrome [46].

The Exscalate4CoV consortium supported by the European Commission’s Horizon 2020 (Grant Agreement N. 101003551) also identified Raloxifene as a good blocker of COVID-19 infection using an Artificial Intelligence platform combined with an HPC approach [47]. These results are in line with others published in [48]. Consequently, AIFA approved a clinical trial to test the efficacy of Raloxifene in mild- or pauci-symptomatic COVID-19-infected patients [49], and a prospective study investigated the effect of Raloxifene therapy and SARS-CoV-2 immunity in vaccinated subjects (RALOXIVAXI). In our study, the number of events related to women undergoing therapy with EMs was too small to allow for a statistical evaluation compared to the number of events related to women who were not undergoing any of the considered therapies. However, among the 11 patients undergoing treatment with EMs, none of them were infected with SARS-CoV-2.

Finally, our results indicate that considering the other treatments, only women treated with vitamin D3 or vitamin D3 and calcium showed a significant reduction in the risk of SARS-CoV-2 infection by 26% (OR 0.74; 95%CI 0.60–0.91) and conversely, an increased risk of hospitalization (OR 2.69; 95%CI 1.77–4.07). The interest in vitamin D supplementation has been high since the beginning of the pandemic; however, despite the large amount of available literature, its role still remains controversial [50,51,52,53,54]. Vitamin D is a steroid hormone that takes part in several physiological processes related to immunity and infections, the cardiovascular system, and bone integrity [55]. Furthermore, the use of vitamin D supplementation in the prevention of respiratory tract infections has already been supported by several clinical trials and systematic reviews [56,57,58].

A higher mortality rate in COVID-19-positive patients has been observed in subjects with a vitamin D deficiency compared to normal levels of vitamin D [50]. However, the sample size and the lower incidence of mortality rates in the cohort of participants render these data insignificant. Furthermore, lower levels of vitamin D have been observed in long COVID patients [59]. Despite the large amount of observational evidence concerning the use of vitamin D in COVID-19 patients, the integration of vitamin D into the guidelines for COVID-19 management has not yet been endorsed by any government agencies or the WHO, mainly due to the heterogeneity of the study characteristics. This underlines the need for a uniform methodology to obtain a final outcome on vitamin D utility [60]. The therapy for osteoporosis management often includes calcium supplementation in association with vitamin D; calcium is an essential mineral element involved in physiological processes, including the immune system, hormone secretion, blood coagulation, etc. [61]. Studies report a high prevalence of reduced calcium levels in patients with a coronavirus infection, showing that patients with higher levels of calcium may be more resistant to this virus than people with hypocalcemia. These works suggest that the maintenance of normal serum calcium levels during a SARS-CoV-2 infection may prevent COVID-19 disease progression [62,63,64,65]. Therefore, it is possible to speculate that the observed protective effect of vitamin D/vit D + calcium supplementation against SARS-CoV-2 infection is related to their important roles in the immune system.

A limitation of this study may derive from the choice of considering all women aged 50 years or more as being in menopause, according to [66,67]. A number of women in the study cohort may not be in menopause yet; however, this proportion is likely limited, with a marginal impact on the study results.

## 5. Conclusions

The findings of this retrospective study, conducted on a population of women undergoing pharmacological treatments for the management of menopausal symptoms with Estrogen Modulators (EMs); Estrogen, Progestin and their combination (EPs); Bisphosphonates (BIs); and vitamin D3 ± calcium (CC) did not reveal any significant impact on infection rates or hospitalizations. However, our results support the protective role of vitamin D3 and calcium supplementation, with a significant reduction in the risk of SARS-CoV-2 infection by 26% (OR 0.74; 95% CI 0.60–0.91), although an increased risk of hospitalization was also observed (OR 2.69; 95% CI 1.77–4.07).

## Figures and Tables

**Table 1:** Demographics and clinical profile of the study cohort.

	Cohort of Women		SARS-CoV-2 Positive		SARS-CoV-2 Negative		*p*-Value
	Number	%	Number	%	Number	%	
Total	124,393	100	6412	100	117,981	100	-
**Age (years)**							
(median, IQR)	64	55–82	75	57–87	63	55–81	<0.0001
**Type of comorbidity ^1^**							
Cardiovascular	32,369	26.0	2121	33.1	30,248	25.6	<0.0001
Neurologic	11,024	8.9	814	12.7	10,210	8.7	<0.0001
Endocrine	9944	8.0	572	8.9	9372	7.9	0.005
Respiratory	5484	4.4	400	6.2	5084	4.3	<0.0001
Psychiatric	5274	4.2	340	5.3	4934	4.2	<0.0001
Rheumatologic	4725	3.8	257	4.0	4468	3.8	0.367
Renal	3217	2.6	215	3.4	3002	2.5	<0.0001
Gastroenterological	2939	2.4	149	2.3	2790	2.4	0.833
Nutrition	1918	1.5	107	1.7	1811	1.5	0.397
Hematologic	1639	1.3	91	1.4	1548	1.3	0.464
Toxic	1322	1.1	63	1.0	1259	1.1	0.520
Others ^2^	2352	1.9	131	2.0	2221	1.9	0.358
**Treatment ^3^**							
None	119,519	96.1	6215	96.9	113,304	96.0	0.004
Bisphosphonates (BIs)	928	0.7	44	0.7	884	0.7	
Estrogens and Progestinic (EPs)	1550	1.2	53	0.8	1497	1.3	
Estrogen Modulators	11	0.0	0	0.0	11	0.0	
Cholecalciferol ± calcium (CC)	2385	1.9	100	1.6	2285	1.9	

^1^ Recorded comorbidities were not mutually exclusive. ^2^ Patients with at least one comorbidity involving infections that were ocular or genetic or with skin disorders or allergies. ^3^ Treatments performed ≤12 months before the date of testing for SARS-CoV-2 infection.

**Table 2:** Risk of SARS-CoV-2 infection, hospitalization, and death in women: multivariable stepwise logistic regression model.

	Infection by SARS-CoV-2		Hospitalization		Death	
	OR	95%CI	OR	95%CI	OR	95%CI
**Age**						
1-year increase	1.021	1.019–1.023	1.018	1.013–1.023	1.095	1.085–1.106
**Type of comorbidity**						
None	1.00	-	1.00	–	1.00	–
Neurologic	1.11	1.02–1.21			1.24	1.00–1.52
Psychiatric	1.11	0.98–1.24	0.83	0.63–1.07	1.22	0.89–1.66
Cardiovascular	0.96	0.90–1.03	0.89	0.77–1.04	0.89	0.74–1.07
Gastroenterological	0.89	0.75–1.05	1.22	0.84–1.76		
Hematologic	0.84	0.67–1.03				
Toxic	0.79	0.60–1.01				
Renal			1.90	1.41–2.57	1.75	1.24–2.46
Rheumatologic			1.52	1.15–2.01	1.44	0.96–2.12
Respiratory			1.51	1.20–1.90	1.32	1.01–1.73
**Treatment**						
None	1.00	–	1.00	–	1.00	–
Bisphosphonates (BIs)	0.80	0.58–1.08	1.23	0.61–2.34	1.08	0.38–2.61
Estrogens–Progestin (EP)	0.88	0.66–1.15	0.83	0.36–1.69	<0.001	−3.01
Estrogen Modulators (EMs)	<0.001	0.00−3.37				
Cholecalciferol ± calcium (CC)	0.74	0.60–0.91	2.69	1.77–4.07	0.80	0.35–1.64

## Data Availability

The dataset used for the analysis will be made available by the authors upon request.

## Supplementary Materials

The following supporting information can be downloaded at: https://www.mdpi.com/article/10.3390/life14091107/s1, Table S1: Absolute number of subjects admitted to hospital and deceased among those infected with SARS-CoV-2, according to type of comorbidity and treatment for menopausal symptoms.
