# Supplementary material for: Retrospective Analysis of the Effect of Postmenopausal Women Medications on SARS-CoV-2 Infection Progression
Source: Life (Basel). 2024 Sep 3;14(9):1107. doi: 10.3390/life14091107 (PMC11433321; doi:10.3390/life14091107)
Supplement: Supplementary file 1 [file life-14-01107-s001.zip › life-3093373-supplementary.pdf]

Supplementary Table 1. Absolute number of subjects admitted to hospital and deceased among those infected with SARS-CoV-2, according to type of comorbidity and treatment for menopausal symptoms

Type of comorbidity	Admitted	Deceased
Respiratory	154	110
Cardiovascular	594	407
Gastroenterological	44	20
Hematologic	31	21
Neurologic	223	178
Psychiatric	83	61
Renal	91	64
Rheumatologic	88	38
Toxic	19	10
Others	251	118
None	666	178
Treatment		
None	1472	704
Bisphosphonates (BI)	13	6
Estrogens-Progestinic (EP)	8	0
Cholecalciferol+-Calcium (CC)	44	8
